# Workplace health promotion for older workers: a systematic literature review

**DOI:** 10.1186/s12913-016-1518-z

**Published:** 2016-09-05

**Authors:** Andrea Poscia, Umberto Moscato, Daniele Ignazio La Milia, Sonja Milovanovic, Jovana Stojanovic, Alice Borghini, Agnese Collamati, Walter Ricciardi, Nicola Magnavita

**Affiliations:** 1 Department of Public Health, Università Cattolica del Sacro Cuore, Rome, Italy; 2 Department of Gerontology, Orthopedics and Neuroscience, Università Cattolica del Sacro Cuore, Rome, Italy; 3 Istituto Superiore di Sanità, Rome, Italy

**Keywords:** Workplace health promotion, Aging workforce, Active ageing, Occupational health, Public health, Lifelong learning, Lifestyle, Frailty, Ageism, Labor policy

## Abstract

**Background:**

Aging of the workforce is a growing problem. As workers age, their physical, physiological and psychosocial capabilities change. Keeping older workers healthy and productive is a key goal of European labor policy and health promotion is a key to achieve this result. Previous studies about workplace health promotion (WHP) programs are usually focused on the entire workforce or to a specific topic. Within the framework of the EU-CHAFEA ProHealth65+ project, this paper aims to systematically review the literature on WHP interventions specifically targeted to older workers (OWs).

**Methods:**

This systematic review was conducted by making a comprehensive search of MEDLINE, ISI Web of Science, SCOPUS, The Cochrane Library, CINAHL and PsychINFO databases. Search terms included ageing (and synonyms), worker (and synonyms), intervention (and synonyms), and health (and synonyms). The search was limited to papers in English or Italian published between January, 1^st^ 2000 and May, 31^st^ 2015. Relevant references in the selected articles were also analyzed.

**Results:**

Of the 299 articles initially identified as relating to the topic, 18 articles met the inclusion criteria. The type, methods and outcome of interventions in the WHP programs retrieved were heterogenous, as was the definition of the age at which a worker is considered to be ‘older’. Most of the available studies had been conducted on small samples for a limited period of time.

**Conclusion:**

Our review shows that, although this issue is of great importance, studies addressing WHP actions for OWs are few and generally of poor quality. Current evidence fails to show that WHP programs improve the work ability, productivity or job retention of older workers. In addition, there is limited evidence that WHP programs are effective in improving lifestyles and concur to maintain the health and well-being of older workers. There is a need for future WHP programs to be well-designed so that the effectiveness and cost-benefit of workplace interventions can be properly investigated.

**Electronic supplementary material:**

The online version of this article (doi:10.1186/s12913-016-1518-z) contains supplementary material, which is available to authorized users.

## Background

In the last 50 years, many industrialised countries have witnessed a change in the age structure of the population [1]. The International Labor Organization has estimated that by 2025, there will be a 32 % increase in the number of people aged over 55 years. Older people will make up approximately 30 % of the population in Europe and North America, 21 % in Asia and 17 % in Latin America. Thus, in Europe, the current demographic trend is towards an increasingly aged workforce [2].

There are several reasons for this phenomenon: first of all the baby boom that followed the Second World War and the low birth rates of the 1980s [3], which together have led to a decline in the inflow of young workers. The increase in life expectancy and the lack of social resources has led most European Governments to raise the retirement age. Additionally, the increased number of older employees in the workforce is due mainly to workers delaying retirement, training for alternative careers and prolonging their longevity thanks to advances in healthcare [4].

The definition of older worker (OW) is still controversial, since different agencies and organizations are using a broad spectrum of ages, ranging from 40 to 65 years, or more. For example, the U.S. Department of Labor, in agreement with most of the literature [5], considers workers to be older if aged 55 years or more [6], whereas the US Age Discrimination in Employment Act [7] provides protection for anyone in the workplace over the age of 40 years. In the workplace sector, workers aged over 45 years are generally considered to be “older” [3].

OWs may differ from their younger counterparts due to a number of physical/biological, psychological/mental, and social characteristics that influence their needs, expectations and challenges [8]. Physical functions, including sensory abilities (e.g. eyesight and hearing), muscular function (e.g. balance, strength and flexibility), aerobic capacity (e.g. VO_2_ max), reaction time and speed, immune response and the ability to maintain homeostasis all decline with age, and this deterioration becomes even more pronounced after the age of 50 [9]. In addition, OWs usually have a higher prevalence of aging-related metabolic disorders such as abdominal obesity, hypertension, hyperglycemia, and dyslipidemia [10]. Consequently, they are often subjected to polypharmacy, with lower quality of life, reduced mobility and mild cognitive impairment, all of which are of concern at the workplace [11, 12]. Bearing in mind that OWs might become more sensitive to changes and less willing to accept them, it is highly probable that the majority will manifest difficulty in adjusting to new job techniques and conditions at the workplace. Moreover, published reports show an increase in perceived age discrimination among OWs which often results in negative feelings, such as uselessness, powerlessness and low self-esteem [13]. Because the ageing workforce has become an increasingly important occupational health issue, especially in relation to a nation’s economic prosperity, maintaining a healthy and productive workforce is a key goal of European labor policy [14].

The worksite is generally a promising setting for health promotion. Theoretically, in the workplace, where employees spend a great amount of time, a number of promotion strategies can be used to provide opportunities for behavioral changes. Furthermore, the occupational health (OH) service, which is part of the work organization, can use its know-how to introduce effective workplace health promotion (WHP) interventions [15], encouraging employees to take own responsibility and stimulating self-help, through participatory ergonomics [16].

Although there has been extensive investigation of WHP programs, these studies, however, are usually focused on the entire workforce [17, 18] and to a specific topic, such as healthy eating [16], or presenteeism [17]. Review studies on OWs, on the other hand, are specifically targeted to a single topic, such as safety and health needs of OWs [19], or OH services in the workplace, [20]. A recent review on the effectiveness of WHP for OWs found only 4 studies on early retirement work ability and productivity [21]. Our aim was to determine whether a wider range of WHP interventions developed specifically for older adults could significantly improve their ability to remain in the workplace and adapt to modern work methods and health conditions.

Although Pitt-Catsouphes et al. [22] claim that the official classification of promotion programs is still inadequate, there appear to be three broad categories: screening activities to identify potential health risks (e.g. ergonomic assessments; health risk assessments, etc.); lifestyle management activities to improve health and to prevent/minimize health risks, including those associated with chronic conditions (e.g. exercise programs; healthy food options in cafeterias, etc.); and on-the-job lifelong-learning interventions that encourage employees to remain in the workforce. Promotion programs should also focus on developing internal policies for OWs so as to address disparities among subpopulations of workers and thus avoid ageism [23, 24].

When ‘healthy ageing management’ is concerned, OH services could probably play a key role in the workplace. One study suggested that policies should look at ageing workers rather than older workers, starting with those as young as 45. It also stated that OH strategies are needed to address the challenges of an ageing workforce, which include the prevention of work-related diseases, diminished work performance due to chronic diseases and the promotion of health and workability [20, 25]. Organizations, both large and small, can engage in this new strategy by systematically integrating their health promotion safety and environmental programs and policies [16, 26]. Furthermore, workplace ergonomics should be age-appropriate and bear in mind the capabilities and limitations of older employees [16]. These concepts are reasonable, but there is still no sufficient evidence that WHP programs for OWs based on these assumptions are attractive to workers and companies.

The purpose of this review was to systematically summarize and scientifically appraise the literature on WHP for OWs, identify the institutions and study groups active in this field and the type of activities performed. This “state of the art” picture might help recognizing and spreading good practices in workplaces.

## Methods

### Search strategy

Comprehensive database searches were performed within the framework of the EU-CHAFEA ProHealth65+ project by two independent investigators to identify potentially relevant articles from MEDLINE, ISI Web of science, SCOPUS, The Cochrane Library, CINAHL and PsychINFO databases. The search strategy, based on the implemented PICO model, was developed first devised for use in MEDLINE and subsequently adapted for the other databases. The combination of keywords used at MEDLINE were: (aging OR ageing OR elderly OR aged OR old) AND (worker* OR workforce OR employee* OR farmer* OR craftsman OR laborer) AND (Health OR safety) AND (promote* OR prevent* OR intervention OR program*) AND workplace AND (Active Ageing and Job OR Well-Being OR work ability OR disability OR impairment OR rehabilitation OR fitness OR capacity OR retention OR re-entry OR employability OR adaptation OR satisfaction OR attitude* OR discrimination OR integration OR productivity OR absenteeism OR presenteeism OR mental health OR stress OR learning OR life style* OR occupational disease*).

### Inclusion and exclusion criteria

Studies were considered eligible if they reported interventions which were conducted specifically on OWs, or at least had the aim to promote healthy/active ageing in the working population. If the intervention was targeted at both the entire workforce and younger workers, only subgroup analyses for ageing workers were deemed eligible. The studies included had to be original research (randomized controlled trials (RCT), quasi-experimental studies (NCT), pre-post, cohort, case-control, ecological and cross-sectional studies), or primary studies derived from relevant systematic reviews and meta-analyses.

Studies that analyzed the clinical course of age and/or work-related illnesses were excluded. The search was limited to papers in English and Italian published between January, 1^st^ 2000 and May, 31^st^ 2015.

### Study selection

After completion of the searches and exclusion of duplicate studies, the initial screening of publications included an independent review of titles and abstracts by two researchers who then had to try to reach agreement on study inclusion. Any discrepancies between researchers were resolved through consensus and, if necessary, by consulting a third reviewer. Full papers were obtained for studies that fitted the inclusion criteria, and each paper was reviewed for quality and data extracted. In addition to the computerized search, references from included studies were also checked (i.e. snowball method) to ensure that no relevant publications had been omitted.

### Data extraction and synthesis

The following information was extracted: first author, year of publication, type of study, study location, workplace, intervention, characteristics of participants, outcome measurements, follow-up periods, and key findings.

The interventions were classified according to their outcomes that were defined in the following domains: 1) Policy for older workers, 2) Job retention, 3) Workability and productivity, 4) Health and well-being. Articles were included in one or more groups according to the main endpoints of the study. The framework described was developed by taking into account information obtained from “Healthy Work in an Ageing Europe”, the 5^th^ Initiative of the European Network of Workplace Health Promotion [27] and other relevant milestones from gray literature [28].

### Quality assessment

The methodological quality of each study was assessed using an Effective Public Health Practice Project (EPHPP) “Quality Assessment Tool for Quantitative Studies” that evaluates six main domains: (1) selection bias; (2) study design; (3) confounders; (4) blinding; (5) data collection method; and (6) withdrawals/dropouts, each of which can be rated as good, mediocre or poor. An overall score was obtained for each study by adding up the separate section rating (see Additional file 1).

The systematic review was conducted in accordance with the Preferred Reporting Items for Systematic Reviews and Meta-Analyses (PRISMA) guidelines.

## Results

**Fig. 1:**
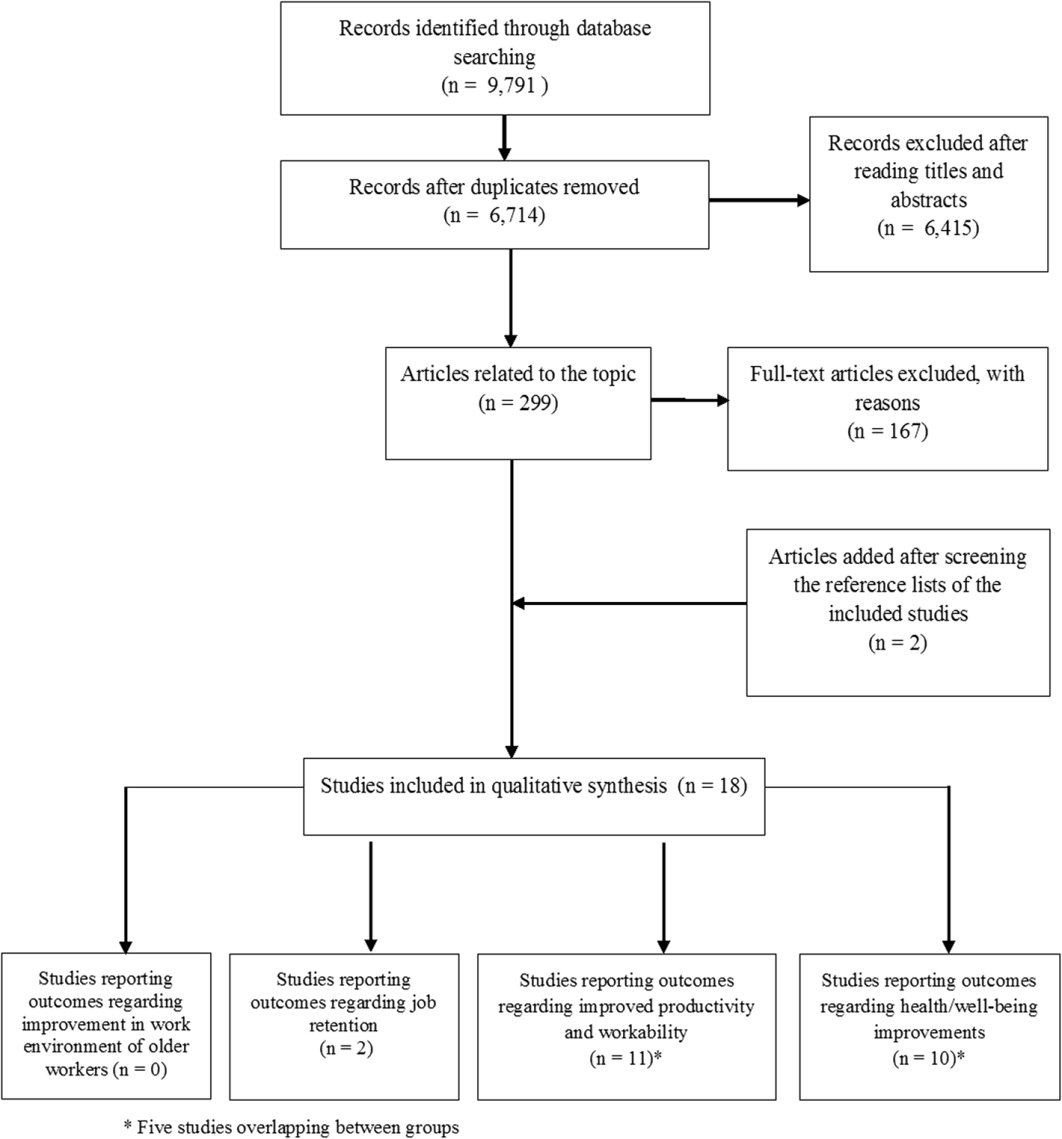
Flow chart

The literature search yielded 9791 papers, 3077 of which were duplicate studies. After initial review of the titles and abstracts, 6415 articles were excluded. Two reviewers independently examined 299 potentially eligible articles. 18 studies [14, 29–45] describing a wide range of promotion interventions were included in the final systematic review (Fig. 1). Five studies [32, 36–39] overlapped domains 3 and 4. The characteristics of each eligible study are illustrated in Table 1.

**Table 1:** Characteristics of each eligible study

Author (year)	Country	Population (*N*)	Intervention (*N*)	Control (*N*)	Follow-Up	Outcome	Main results	Study design and quality
DOMAIN 2-Increasing job retention								
Wallen & Mulloy (2006) [29]	USA	50 Factory workers at a medium sized electronics manufacturing plant	Computer-based respiratory safety trainings to young (<44 yrs) and older (44+ yrs; mean age 51 yrs) workers	Comparison of 3 different programs: TXT, TAP and NAP	–	Learning evaluation	Older participants who received instructions with NAP reached significantly higher level of learning than TXT/TAP participants; older learners may benefit from this approach	Cross Sectional Weak
McDonald et al. (2010) [30]	Australia	27 Hospital Nurses	Aged nurses acting as mentors (12 nurses aged between 40 and 70) and young mentees (15)	–	6 months	Ideas and perceptions of mentors collected by interviews	Mentoring programmes, in particular including retirees, can stimulate professional development, personal growth and benefits both in mentors both in mentees.	Collective case study NA
DOMAIN 3-Improving productivity and workability								
Karazman et al. (2000) [31]	Germany	122 Tram, bus and subway drivers of the Munich Transportation Authority 45 and older	20 health days training (physical exercise, professional skills training and self-experience)	–	1 year	WAI (stratified according another specific tool-effect typology questionnaire)	Non-significant increase of WAI (except in a subgroup of older participants)	Pre-post Weak
Mackey et al. (2011) [36]	Australia	Academic and administrative employees between 45 and 70 (mean age = 54 yrs) without a physically active lifestyle	12 weeks long flexible, individually targeted walking intervention facilitated by an occupational physiotherapist (32)	Usual activity (32)	12 weeks	Measures for work ability	This worksite based intervention using behaviour change principles produced significant improvements in work ability	RCT (in press) NA
Rutanen et al. (2014) [40]	Finland	123 occupationally active symptomatic menopausal women aged 44–62 (mean age 54)	6 months aerobic exercise intervention 4 times a week, 50 minutes per session, with a progressive increase in intensity (60)	Without intervention (63)	24 weeks	WAI and Questionnaires on the daily physical and mental work strain	Women in the intervention group have lower work strain, but a tendency towards higher WAI.	RCT; Moderate
Koolhaas et al. (2015) [32]	Netherlands	Nurse and administrative personnel (workers and supervisors) from a University and a University Medical Center (mean age 52)	“Staying healthy at work” problem-solving based intervention to achieve improvement, life-long learning or to tackle problems for a sustainable working life. (64)	Usual activity (61)	1 year	WAI and Productivity (the Quality and Quantity method)	None or negative effects were found on main outcomes (respectively on productivity and WAI). However, effectiveness was shown on three of the secondary outcome measures (work attitude, self-efficacy and skill discretion)	NCT; Moderate
De Boer et al. (2004) [33]	Netherlands	116 employees older than 50 years (mean age 53 yrs) at risk for early retirement of a large international company, which develops and manufactures electronic equipment.	Construction of a detailed action plan, consultation of the employee’s supervisors and personnel managers, and, if appropriate, referral to the general practitioner, a medical specialist, or psychologist. (61)	Received care as usual (they were not invited for a consultation but they could always consult their occupational physician on request) (55)	2 years	Work Ability Index, the Utrecht Burn Out Scale, and the Nottingham Health Profile measuring quality of life	After 2 years no significant differences (except for burnout) in work ability, quality of life and early retirement in the intervention group. A significant improvement was found in the mid term analysis.	RCT; Strong
Palumbo et al. (2012) [37]	USA	14 Nurses older than 49 years (mean age 54 yrs) at one academic medical center	On-site Tai Chi classes once a week and practice on their own for 10 minutes each day for 15 weeks. (7, but 6 included in the analysis)	No intervention (7, but 5 included in the analysis)	15 weeks	Productivity	Tai chi group showed a significant improvement in work productivity (+3 %) and seems cost savings (preliminary cost analysis)	RCT; Moderate
Strijk et al. (2013) [39]	Netherlands	Older workers (45 years or older-mean age 52 yrs) from two academic hospitals	The Vital@Work group (367 workers) received a 6-month Vitality Exercise Program, Personal Vitality Coach visits, and free fruit.	No intervention written except information about a healthy lifestyle in general (363 workers)	1 year	Work engagement, productivity and sick leave	No significant differences regarding work engagement, productivity and sick leave were observed	RCT; Moderate
van Dongen et al. (2013) [38]	Netherlands	See-Strijk et al. (2013) [39]-Domain 3			1 year	Costs related to the Vital@Work intervention, Health care utilization, sport, absenteeism and presenteeism	The program was neither cost-effective (COI) nor cost-saving (ROI).	A COI and ROI analysis of Strijk et al. (2013) [39] RCT: NA
Siukola et al. (2011) [14]	Finland	Blue-collar workers aged 55 years or older (mean age 57 yrs) from Finnish food company	A senior programme looking at the specific needs of older worker with work-related arrangements and dispensations (opportunities to alter the content of work, need for rehabilitation or education) (129)	No intervention (229)	6 years	Total sickness absence days and spells of 1–3, 4–7, 8–21 and >21 days	Sickness absence days increased significantly from baseline in both groups. Intervention group had higher risk for short-time sickness absence, with a reduced risk of long-lasting one	NCT; Weak
Goine et al. (2004) [34]	Sweden	Two paper and pulp manufacturing plants. No specific intervention for older worker, but analysis were stratified for 50–59 and 60–64 age classes	PLANT A (1200) implemented an extensive programme of managerial training and vocational rehabilitation activities. It received about four times more financial support than plant B	PLANT B (1600) Without implementing programmes and with less financial support	10 years (1989–98)	Sick leave and disability pensions.	For employees in the upper age groups, relative risk for long-term and very long-term sick leave was and remained elevated after the intervention. The RR of short-term sick leave (1–14 days), was (not significantly) lower in these groups than among the younger employees.	Cohort; Moderate
Härmä et al. (2006) [35]	Finland	Line maintenance unit of a large airline company	Implementation of a very rapidly forward rotating workplace shift system among young (24–44 years) and elderly (45–61 years) maintenance workers	Without intervention	2 years (1.5 years before and 6 months after a new shift system	Sleep wakefulness, well-being and social life of young and older shift workers	The intervention had positive effects on the sleep, alertness and well-being (including social and family life and hobbies), especially for the older shift workers	NCT; weak
DOMAIN 4-Workplace interventions for health promotion and well-being								
Strijk et al. (2012) [43]	Netherlands	Older workers (45 years or older-mean age 52 yrs) from two academic hospitals	The Vital@Work group (367 workers) received a 6-month Vitality Exercise Program, Personal Vitality Coach visits, and free fruit.	No intervention written except information about a healthy lifestyle in general (363 workers)	6 months	Lifestyle behaviours (sports, vigorous physical activities and fruit intake) and vitality-related outcomes (aerobic capacity, mental health and the need for recovery after a work day)	The intervention favourably affected the weekly sports activities, the fruit intake and the need for recovery No effects were observed for other outcomes.	RCT; Moderate
Strijk et al. (2013) [39]	Netherlands	See-Strijk et al. (2013) [39]-Domain 3			1 year	The primary outcome was Vitality (the RAND-36 vitality scale for general vitality, and UWES for work-related vitality)	No intervention effects were observed for vitality, even if high yoga compliers significantly increased their work-related and general vitality.	RCT; Moderate
Palumbo et al. (2012) [37]	USA	See-Palumbo et al. (2012) [37]-Domain 3			15 weeks	Several measures for physical and mental health, work-related stress	Tai chi group showed a significant improvement in physical functions and seems (preliminary cost analysis) cost savings	RCT; Moderate
van Dongen et al. (2013) [38]	Netherlands	See-Strijk et al. (2013) [39]-Domain 3			See-van Dongen et al. (2013) [38]-Domain 3			
Chen et al. (2014) [44]	Taiwan	108 Workers aged 50+ years (mean age 55 yrs) from small-and medium scale enterprises	Phase I (4 weeks): organizing action groups, individualized planning of behavioral changes, and updating workers’ health knowledge; Phase II (follow-up 20 weeks) emphasized carrying out the planned lifestyle improvements to reduce the risk of metabolic disorders (58)	Without intervention (50)	24 weeks	Major outcomes were changes in lifestyle, anthropometric and blood biochemical variables	The intervention had a significant positive effect on waist circumference, body weight, BMI, physical activity, triglycerides ad HDL-C. However, the intervention did not improve blood pressure, or serum lipid or HbA1c levels, vegetable consumption, time use, or sleep duration, nor the proportions of subjects having metabolic disorders. The control group had a significant time-related decrease in total cholesterol and HDL-C	NCT; Strong
Merrill et al. (2011) [42]	USA	440 young (18–49 yrs) and old (50+ yrs) workers in a small company. Stratification according to age class allow specific analysis for 64 older worker	All employees receive a four-level wellness programs and quarterly screenings, with prizes and incentives for participants.	No Control	3 years (2007–2009)	Selected Health Indicators: blood pressure, flexibility, body fat, body weight	Overall positive effects. Older employees, who had the highest blood pressure and weight at baseline, showed the greatest decreases in blood pressure and weight.	Cohort; Moderate
Mackey et al. (2011) [36]	Australia	See Mackey et al. (2011) [36]-Domain 3				Measures for step count, % body fat, waist circumference, blood pressure, physical activity & psychological wellbeing	This worksite based intervention using behaviour change principles produced significant improvements in physical activity and health status	RCT (in press) NA
Hughes et al. (2011) [41]	USA	423 participants (older support and academic staff at the University of Illinois at Chicago) aged 40 years and older (mean age 51, range 40 to 68) were categorized into 3 study arms	The COACH (150 workers received a Web-based risk assessments with personal coaching support); the RealAge (135 workers received only Web-based risk assessment and behaviour-specific modules)	The control group received printed health-promotion materials (138 workers)	1 year	Dietary behaviours; Physical activity; Stress. Smoking cessation; Body mass index, waist circumference, and weight.	In the COACH group significant amelioration in fruits and vegetables consumption, percentage of energy derived from fat and physical activity. RealAge participants experienced a significant decrease in waist circumference COACH group participants were almost twice as likely to use their intervention as RealAge participants used theirs.	RCT; Moderate
Cook et al. (2015) [45]	USA	50 years of age and older (range 50–68 yrs) employees located in multiple US offices of a global information technology company (278)	HealthyPast50 workers received a Web-based multimedia program containing information and guidance on the major health promotion topics (138)	wait-list control condition (140)	3 months	Measures of healthy aging, diet, physical activity, stress management, and tobacco use	The HealthyPast50 group performed significantly better than the control group on diet behavioural change self-efficacy, planning healthy eating, and mild exercise. There were not significant improvements on eating practices, moderate exercise, and overall exercise.	RCT; Weak
Koolhaas et al. (2015) [32]	Netherlands	See Koolhaas et al. (2015) [32]-Domain 3				Vitality (the single-item vitality scale of the 12-Item Short Form Health Survey)	Negative effects were found on Vitality. Workers in the intervention group had a 0.10 times higher odds of being in a higher vitality category than the persons in the business as usual group.	NCT; Moderate

*Abbreviations*: *NA* Not Applicable; *TXT* text only, *TAP* text with pictures, *NAP* text with pictures, and audio narration, *RCT* Randomized Controlled Trial, *NCT* Non-Randomized Controlled Trial, *WAI* work ability index, *COI* Cost of Illness, *ROI* Return on Investment

The study populations came from different geographical sources: ten were European studies (five were conducted in the Netherlands, three in Finland, one in Germany and one in Sweden), five were North American (USA) studies, two were conducted in Australia and one in Taiwan.

Considerable variation was found both in the workplaces described and in the age used to define the “older” workers who participated in the studies. Most authors considered employees of 40/45 years to be older workers, although the mean age of study participants usually ranged from 51 to 55 years. The widest age range was reported by McDonald who referred to nurses between 40 and 70 years as mature age mentors.

As regards study design, eight studies were randomized controlled trials (RCTs), and four non randomized controlled trials (NCTs); there were also two cohort studies, one cross-sectional study, one pre-post study, one cost-effectiveness analysis, and one collective case study. Selected studies differed considerably in methods and type of intervention, outcomes and follow-up periods.

EPHPP analysis of interventions, based on study design, selection bias, randomization criteria, confounders, blinding, withdrawals and drop-outs, intervention integrity, and analyses revealed that 2 out of the 18 studies were of good overall quality, while 8 were mediocre and 5 were of poor quality (3 studies were not classifiable).

- Domain 1-Policy for older workers
  - Initially the review focused on identifying interventions that addressed the development of policies for OWs, aimed at improving interpersonal communication between the latter and other workplace employees, or combatted the exclusion or discrimination of OWs. Only a few [46–54] narrative studies were found to refer to the risk of ageism or express the need for a policy for OWs in the workplace, but since none of these described any intervention, they were not included in the review.
- Domain 2-Increasing job retention
  - In the USA, Wallen and Mulloy [29] evaluated the response of a small sample of electronic company workers to a computer-based respiratory safety-training program. Three versions of the program (text, text with pictures, text with pictures and audio narration) were shown to employees who then took a high- and a low-level learning test. Younger workers (under 44 years of age) did better overall. No significant effects of age or treatment were observed on low level learning, while workers over the age of 45 years improved in the high-level learning test only after computer-based training with pictures and audio narration. McDonald et al. [30] proposed a mentoring service for nurses provided by a small group of older and retired nurses. Implementation of this program brought benefits for both mentors and mentees since it produced positive effects in three main areas by facilitating work and life decisions, by visibly helping other nurses and midwives, and by adapting to the role and the mentee. The mentors were a valuable source of knowledge; they also helped their mentees to manage and enjoy a long-term nursing career and cope with the high demands of the work environment. Moreover, they promoted the professional development and personal growth of mentees.
- Domain 3-Improving productivity and workability
  - 11 studies [14, 31–40] reported interventions aimed at improving work ability, work organization and productivity in OWs, and at postponing the prospect of early retirement. However, a real change in work organization was the main experimental intervention in only 5 of these studies [14, 32–35]; most of the studies explored the effect of various physical training programs on the aforementioned outcomes [31, 36–40].
  - 3.1 Workplace interventions for maintaining work ability and postponing early retirement
    - Most studies [31–33, 36, 40] used the work ability index (WAI) as a way of self-assessing individual ability to deal with work demands. Karazman et al. [31] reported intervention in a subgroup of older participants selected through the “effect typology” questionnaire that identifies specific psychobiological patterns of response to intervention. Results from this study yielded a non-significant increase in the WAI after a 1-year health promotion program based on physical, psychological and stress management training accompanied by diet counselling. However, the authors highlighted a salutogenic effect of OH promotion intervention, due to an increase in the WAI and a decline in the desire for early retirement.
    - Mackey et al. [36] investigated the possible effects on workability of a 12-week individually targeted walking program. Preliminary results indicated that this kind of worksite based intervention, individually tailored for OWs, can produce significant improvements in physical activity and, it is hoped, in work ability.
    - Similarly, Rutanen et al. [40] investigated the effects on work ability and strain among menopausal female workers of a 24-week physical exercise program. At the end of this intervention, physical strain was lower in the treated group than in controls; however, differences in the WAI were not significant.
    - The study by Koolhaas et al. [32] assessed intervention aimed at creating a motivating and healthy work environment through the use of problem-solving techniques. The program failed to produce effects on productivity, but had a significant, negative effect on the WAI. Nevertheless, the program was shown to be effective with regard to some secondary outcomes such as work attitude, self-efficacy, and skill discretion.
    - In a very small self-selected group of OWs from an electronic company, intervention that included consultation with supervisors, the development of an action plan and referral to medical care when appropriate proved to be effective in reducing the frequency of early retirement after a short period (6 months). On the other hand, the overall rate of retirement (including disability retirement) in the intervention group was similar to controls at the end of the follow-up [33].
  - 3.2 Workplace interventions for improving work organization and productivity
    - A limited number of studies reported interventions that focused on productivity, absenteeism, sickness absence and presenteeism in OWs.
    - A pilot study was conducted by Palumbo et al. [37] on a very small group of workers (6 nurses) to evaluate the feasibility of a Tai Chi workplace wellness program. Most of the results failed to show statistically significant group differences in changes over time.
    - In the Vital@Work study, a 6-month lifestyle intervention in the workplace that aimed to increase older workers’ productivity and decrease sick leave by improving mental (e.g. via yoga sessions) and physical (e.g. via aerobic exercising) vitality factors, failed to show any significant differences between cases and controls [39]. Subsequent cost-benefit analysis [38] failed to reveal any significant positive findings related to absenteeism and presenteeism, thus indicating that this program was neither cost-effective (from a societal point of view) nor cost-saving (for employers).
    - In a Finnish food company, Siukola et al. [14] introduced a program for OWs designed to increase workability and the willingness to work until age-based retirement. A significant increase in the median number of sickness absence days per person/year was reported for the intervention and control groups during the follow-up period. Compared to the control group, the intervention group had a higher odds for short-term sickness absence, with a reduced odds of long-lasting sickness absence.
    - Only one study was designed to evaluate the effect on the personal and relational life of workers of an organizational intervention involving the implementation of a very rapid forward rotating workplace shift system [35]. The authors concluded that the ergonomic change had positive effects on sleep, alertness and well-being (including social and family life and hobbies), especially in the older shift workers.
- Domain 4-Workplace interventions for health promotion and well-being
  - 10 studies [32, 36–39, 41–45] reported interventions aimed at promoting health and lifestyle changes or reducing the risk of ill health in OWs.
  - The main outcome assessment made in the aforementioned Vital@Work study [43] concerned vitality. After the initial 6-months of follow up, the authors observed positive, statistically significant effects on sports activities and fruit intake, as well as on the need for recovery after a work day. However, no improvements were observed in vigorous physical activity, aerobic capacity or mental health. Furthermore, at 12-month follow up, no effects of intervention were observed for the main outcome, although there was a significant increase in the work-related and general vitality of the subgroup of high yoga compliers.
  - Similar, though non statistically significant results, were reported for the Tai Chi workplace wellness program in the aforementioned study of Palumbo et al. [37].
  - WHP programs were also implemented in small and medium-sized companies. A 24-week intervention trial designed to improve lifestyle, team spirit and goal keeping in workers aged 50+ years from small and medium scale enterprises in Taiwan significantly reduced workers’ waist circumference, body weight and BMI [44]. However, this intervention did not improve blood pressure, serum lipid, or HbA1c (glycated haemoglobin) levels, vegetable consumption, time use, or sleep duration. Merrill et al. reported an experiment in medium-sized companies [42] that involved promoting physical activity, better nutrition, smoking cessation, and health education seminars. All workers were involved in quarterly monitoring. Retrospective analysis of 3 years of activity showed overall positive effects. Employees aged 50 or older, who had the highest blood pressure and weight at baseline, showed the greatest decrease in these parameters after the follow-up period.
  - Some interventions were based on specifically designed websites. A web-based multimedia program containing information and guidance on the major health promotion topics of healthy aging, diet, physical activity, stress management, and tobacco use, proved to be effective in obtaining behavioral modifications in mature aged workers [45]. The availability of personal coaching support in addition to web-based health risk assessment significantly increased the effectiveness of a WHP program [41].

## Discussion

To the best of our knowledge, this is the first systematic review specifically devoted to investigating and assessing the quality level of studies regarding WHP programs for OWs This paper updates the evidence provided by previously published reviews about health promotion and well-being in OWs [18–20], but also includes other relevant domains of a comprehensive WHP approach (job retention and productivity/workability). Our study shows that, although this subject is of great importance, there are only a few studies that evaluate WHP actions for OWs. The literature on recent interventions to improve the working climate, train older workers, provide ergonomic conditions at the workplace and promote or maintain health in the aging workforce is rather limited. The WHP programs are aiming different areas: physical exercise, eating habits, walking, etc. It may be that different programs need different time to show an effect. For example: people may get fitter much faster with exercise program than changing their beliefs. Unfortunately, most of the available studies have been conducted on small samples, for a limited period of time, with methods the same author sometimes described as unsatisfactory. Evidence of effectiveness is often lacking, especially in the long term. The considerable variability in methods, standards and outputs, in addition to the overall mediocre quality of most of the studies, make it difficult to draw conclusions.

Studies on the training of older workers have usually been conducted on very small convenience samples, making it difficult to generalize results. However, the finding that learning in OWs differs from that in younger employees, and that it benefits from a multimodal approach [29] is in agreement with the general orientation of the literature [55]. Likewise, there is no reason to believe that the experience described by McDonald et al. [30], in which OWs were used as mentors, cannot be repeated successfully in other companies. As the authors claim, by respecting and enhancing diverse points of views and experiences, mentoring initiatives offer an important intergenerational and cross-cultural opportunity to facilitate the creation of a learning environment and trusting relationships.

An analysis of the actions that have attempted to change work ability, absenteeism, or retirement intentions, inevitably reveals a discrepancy between resources and objectives. WHP interventions based on short-term physical exercise generally failed to improve the work ability of workers [31, 32, 40], or at best obtained a transient improvement that soon disappeared [33]. The observation period was usually too short to ascertain whether these programs were able to at least prolong previous ability. Similar conclusions can be reached for the studies that aimed at reducing early retirement [31], absenteeism or presenteeism [37, 38]. In some cases the observed effect was of dubious benefit [14], since it produced only a shift in the pattern of absences, not a reduction in absenteeism. The authors’ statement, that the increase in short-term absence (so-called “compensatory absence”) can be linked to a reduction in early retirement, is controversial. In fact previous studies have demonstrated that workers who make use of short-term absences, have a high level of self-perceived job strain and a low level of social support at work [56]. Although in the planning and pilot phases of the studies the attitude of researchers was always optimistic about the possibility of significantly improving the health and productivity of OWs [36, 37], after the follow-up period, results were often questionable, or even negative [38]. On examining these findings we cannot definitively rule out the possibility that paradoxal results, i.e. the worst effects seen in the intervention group, could be due to the small number of observations or unsatisfactory epidemiological methods. Even in our search we came across a large number of experiences and recent reviews on this issue [20], we confirmed a lack of longitudinal research and studies of high quality. Additional intervention studies are needed to support evidence-based decision for ageing workers.

Programs aimed at maintaining the health and general well-being of OWs varied greatly across studies and seemed to be much more effective than those focused on changing workplace conditions or improving work ability and productivity. The most common interventions included physical activity training, such as aerobic exercises, yoga or Tai Chi courses, walking programs, etc. We must confirm, however, that there is still a large number of research gaps, including the lack of longitudinal studies on some relevant disorders, such as stress and anxiety, musculoskeletal disorders, accidents, and suicide risk in OWs, as previously observed [18].

In general, we may observe thatWHP is thriving in the USA, while European studies are limited to the experiences of a few research groups, mainly in Northern countries. One of the reasons for this is that in many European countries the cost of employee sickness absence is generally incurred by the NHS (National Health Service), and this does not encourage companies to spend on health prevention [57], or provide information for educative purposes and offer self-help programs. Another important factor is that the health and safety of workers traditionally follows a labor approach: prevention only applies to aspects related to work, while general health protection and promotion (e.g. health related to life-style) are not included in this framework [58]. Moreover, as the increase in the age limit for retirement is a very recent innovation in all European countries, there has not been enough time to prepare and implement promotion programs specifically targeted at workers over 55 years of age. Finally, it is clear that, from the point of view of companies that intend to promote the health of their workers, intervention should not be limited to older workers alone, but should ensure that there is a beneficial effect for all employees. Employers who provide WHP programs make an investment in human capital; they rightly expect to have a return over time, not only in terms of health, but also in productivity and product quality as shown in previous studies on the whole workforce [59]. The sensitivity of companies towards health promotion does not depend on size, total income or other econometric parameters, but mainly on corporate culture. Workplace actions for health are often supported by medium-sized or small companies, with some studies showing the best results in the older workforce [42].

The workplace is an ideal setting for implementing health promotion activities [60–62] because there is greater access to workers in a controlled environment through existing channels of communication and social support networks, e.g. OH Services that facilitate the creation of a supportive culture [63]. The worksite is also a cost-effective setting for providing health education and promoting health behavior change [64, 65]. However, our research confirms the observation of McDermott et al. [20] that very few OH interventions have addressed the health and workability of OWs. There is a wide space to improve OH commitment and account the needs of the older workforce. Our review confirms that WHP interventions are seen as positive by older workers but it is important to ensure equal access to all workers in such promotions, as previously suggested [18].

Our study has some limitations. The heterogeneity of the studies we have included makes it difficult to perform a synthesis of the literature, and the low quality of most of the studies weakens the evidence obtained. Interventions conducted on small convenience samples cannot be applied to the entire working population. Moreover, as in the majority of reviews, publication bias might be an issue.

In conclusion, the insufficient and limited evidence available for a favourable effect of WHP programs is mainly due to a scarcity of RCTs and inconsistent findings between the limited number of studies. The aging of the workforce is a very recent phenomenon and this has so far not allowed the publication of prospective studies of high quality.

Although there seems to be a number of studies that report WHP interventions, especially in the grey literature, the results of the present review emphasize the need for high-quality studies, with follow-up periods that would help researchers to ascertain the effects of intervention and complement them by performing cost/benefit analyses. Besides measuring cost effectiveness of WHP interventions for individuals and their economic impact on the companies, ethical aspects should also be taken into account in the evaluation process. At the moment, the paucity of studies on WHP for OWs prevents a detailed analysis of the relevant bioethical and social issues connected to the problem. In a resource-limited and employment-limited system, making efforts to improve the employability of OWs undoubtedly might decrease the employment of younger ones. In our opinion, rather than thinking about improving the conditions of an age category, we should improve the working conditions of all. Improving health, safety and welfare of workers is a benefit for the whole society. We think this topic may be a good argument for future research.

## Conclusions

On the whole, the evidence currently available does not unequivocally support the effectiveness of WHP interventions targeted at OWs in reducing sickness absence, presenteeism, or the intention to retire in this population. Also there is insufficient evidence that worksite programs can increase the working capacity of older employees. However, there is moderate evidence that active workplace intervention reduces waist circumference, body weight, BMI and other components of metabolic syndrome, and limited evidence that web-based programs may be useful in changing worker’s behavior. Older workers’ health can therefore be improved with active workplace interventions, but careful consideration must be given to the content, quality and cost-effectiveness of this type of intervention.

## Abbreviations

BMI, body mass index; EPHPP, Quality Assessment Tool for Quantitative Studies; HbA1c, glycated haemoglobin; NCTs, non randomized controlled trials; OH, occupational health; OW, older worker; PRISMA, Preferred Reporting Items for Systematic Reviews and Meta-Analyses; RCTs, randomized controlled trials; VO2 max, maximal oxygen consumption, maximal oxygen uptake, peak oxygen uptake or maximal aerobic capacity; WAI, work ability index; WHP, workplace health promotion.

## Additional files

Additional file 1: Quality assessment of the included studies. (XLSX 17.0 KB) Additional file 2: PRISMA statement for reporting systematic reviews. (DOCX 30.4 KB)

## References

[1] Granville G, Evandrou M (2010). Older men, work and health. Occup Med (Chic Ill).

[2] European Commission - Directorate-General for Economic and Financial Affairs. The 2015 Ageing Report. Underlying Assumptions and Projection Methodologies. EUROPEAN ECONOMY 8-2014. http://ec.europa.eu/economy_finance/publications/european_economy/2014/pdf/ee8_en.pdf. Accessed 12July 2016.

[3] Ilmarinen J (2001). Aging workers. Occup Environ Med.

[4] Delloiacono N (2015). Musculoskeletal safety for older adults in the workplace. Workplace Health Saf.

[5] Loeppke RR, Schill AL, Chosewood LC, Grosch JW, Allweiss P, Burton WN (2013). Advancing workplace health protection and promotion for an aging workforce. J Occup Environ Med.

[6] CDC National Center for Chronic Disease Prevention and Health Promotion. Older Employees in the Workplace. Issue Brief No. 1, July 2012. http://www.public-health.uiowa.edu/hwce/wp-content/uploads/2014/11/Issue_Brief_No_1_Older_Employees_in_the_Workplace_7-12-2012_FINAL508.pdf. Accessed 15 September 2015.

[7] The U.S. Equal Employment Opportunity Commission (EEOC). The Age Discrimination in Employment Act. Pub. L. No. 90-202. https://www.eeoc.gov/laws/types/age.cfm. Accessed 12 July 2016.

[8] Brooke E, Goodall J, Handrus M, Mawren D (2013). Applying workability in the Australian residential aged care context. Australas J Ageing.

[9] Ross D (2010). Ageing and work: an overview. Occup Med (Lond).

[10] Bonomini F, Rodella LF, Rezzani R (2015). Metabolic syndrome, aging and involvement of oxidative stress. Aging Dis.

[11] Abraha l, Cruz-Jentoft A, Soiza RL, O'Mahony D, Cherubini A. Evidence of and recommendations for non-pharmacological interventions for common geriatric conditions: the SENATOR-ONTOP systematic review protocol,”. BMJ Open. 2015; vol. 5, no. 1. 10.1136/bmjopen-2014-007488 PMC4316555 25628049

[12] Knoche K, Sochert R, Houston K. Promoting healthy work for workers with chronic illness: A guide to good practice. European Network for Workplace Health Promotion (ENWHP), 2012. http://www.enwhp.org/uploads/media/ENWHP_Guide_PH_Work_final.pdf. Accessed 12 July 2016.

[13] Furunes T, Mykletun RJ (2010). Age discrimination in the workplace: validation of the Nordic Age discrimination scale (NADS). Scand J Psychol.

[14] Siukola A, Virtanen P, Huhtala H, Nygård CH (2011). Absenteeism following a workplace intervention for older food industry workers. Occup Med (Chic Ill).

[15] Greco E, Osnato OU, Magnavita N. Fabbisogni formativi per l’esercizio del ruolo di Medico Competente. IAS Istituto per gli Affari Sociali, Roma, 2015. http://www.safersrl.it/risorse/100528_IAS_fabbisogni_formativi_MC.pdf. Accessed 12 July 2016.

[16] Magnavita N. Engagement in health and safety at the workplace: a new role for the occupational health physician. In: Graffigna G. (eds) “Promoting Patient Engagement and Participation for Effective Healthcare Reform” IGI Global, Hershey, Pennsylvania, 2016.

[17] Maes L, Van Cauwenberghe E, Van Lippevelde W, Spittaels H, De Pauw E, Oppert J-M (2012). Effectiveness of workplace interventions in Europe promoting healthy eating: a systematic review. Eur J Public Health.

[18] Cancelliere C, Cassidy JD, Ammendolia C, Côté P (2011). “Are workplace health promotion programs effective at improving presenteeism in workers? a systematic review and best evidence synthesis of the literature.,”. BMC Public Health.

[19] Crawford JO, Graveling RA, Cowie HA, Dixon K.The health safety and health promotion needs of older workers. Occup Med (Lond). 2010;60(3):184-92. doi:10.1093/occmed/kqq028. 10.1093/occmed/kqq028 20423949

[20] McDermott HJ, Kazi A, Munir F, Haslam C. Developing occupational health services for active age management. Occup Med (Lond). 2010;60(3):193-204. doi:10.1093/occmed/kqq026. 10.1093/occmed/kqq026 20423950

[21] Cloostermans L, Bekkers MB, Uiters E, Proper KI.The effectiveness of interventions for ageing workers on (early) retirement, work ability and productivity: a systematic review. Int Arch Occup Environ Health. 2015 ;88(5):521-32. doi:10.1007/s00420-014-0969-y. 10.1007/s00420-014-0969-y 25118618

[22] Pitt-Catsouphes M, James JB, Matz-Costa C (2015). Workplace-based health and wellness programs: the intersection of aging, work, and health. Gerontologist.

[23] Wegman D.H, McGee J.P. Health and Safety Needs of Older Worker: Report by Committee on the Health and Safety Needs of Older Workers. 2004. Washington, D.C.: The National Academies Press; doi:10.17226/10884. 25032302

[24] Dordoni P, Argentero P (2015). When Age stereotypes are employment barriers: a conceptual analysis and a literature review on older workers stereotypes. Ageing Int.

[25] Ilmarinen J. The ageing workforce--challenges for occupational health. Occup Med (Lond). 2006;56(6):362–4. 10.1093/occmed/kql046 16931565

[26] Hymel PA, Loeppke RR, Baase CM, Burton WN, Hartenbaum NP, Hudson TW, McLellan RK, Mueller KL, Roberts MA, Yarborough CM, Konicki DL, Larson PW. Workplace health protection and promotion: a new pathway for a healthier--and safer--workforce. J Occup Environ Med. 2011;53(6):695–702. doi:10.1097/JOM.0b013e31822005d0. 10.1097/JOM.0b013e31822005d0 21654443

[27] Morschhäuser M, Sochert R. Healthy Work in an Ageing Europe. Strategies and Instruments for Prolonging Working Life. ENWHP European Network for Workplace Health Promotion. Essen, 2006. Accessible at: http://www.ageingatwork.eu/resources/health-work-in-an-ageing-europe-enwhp-3.pdf.

[28] Sitko SJ, Kowalska- Bobko I, Mokrzycka A, Zabdyr-Jamróz M, Domagała A, Magnavita N, Poscia A, Rogala M, Szetela A, Golinowska S. Institutional analysis of health promotion for older people in Europe. Concept and research tool. BMC Health Service Research, 2016 (in press). 10.1186/s12913-016-1516-1 PMC5016730 27608828

[29] Wallen ES, Mulloy KB (2006). Computer-based training for safety : comparing methods with older and younger workers. J Safety Res.

[30] McDonald G1, Mohan S, Jackson D, Vickers MH, Wilkes L. Continuing connections: the experiences of retired and senior working nurse mentors. J Clin Nurs. 2010;19(23-24):3547-54. doi:10.1111/j.1365-2702.2010.03365.x. 10.1111/j.1365-2702.2010.03365.x 20964745

[31] Karazman R, Kloimüller I, Geissler H, Karazman-Morawetz I (2000). Effects of ergonomic and health training on work interest, work ability and health in elderly public urban transport drivers. Int J Ind Ergon.

[32] Koolhaas W, Groothoff JW, de Boer MR, van der Klink JJ, Brouwer S. Effectiveness of a problem-solving based intervention to prolong the working life of ageing workers. BMC Public Health. 2015;15:76. doi:10.1186/s12889-015-1410-5. 10.1186/s12889-015-1410-5 PMC4323226 25648750

[33] de Boer AG, van Beek JC, Durinck J, Verbeek JH, van Dijk FJ. An occupational health intervention programme for workers at risk for early retirement; a randomised controlled trial. Occup Environ Med. 2004 ;61(11):924-9. 10.1136/oem.2003.009746 PMC1757884 15477286

[34] Goine H, Knutsson A, Marklund S, Karlsson B (2004). Sickness absence and early retirement at two workplaces—effects of organisational intervention in Sweden. Soc Sci Med.

[35] Härmä M, Tarja H, Irja K, Mikael S, Jussi V (2006). A controlled intervention study on the effects of a very rapidly forward rotating shift system on sleep—wakefulness and well-being among young and elderly shift workers. Int J Psychophysiol.

[36] Mackey MG, Bohle P, Taylor P, Di Biase T, Mcloughlin C, Purnell K (2011). Walking to wellness in an ageing sedentary university community: design, method and protocol. Contemp Clin Trials.

[37] Palumbo MV, Wu G, Shaner-Mcrae H, Rambur B, Mcintosh B (2012). Tai Chi for older nurses : a workplace wellness pilot study. Appl Nurs Res.

[38] van Dongen JM1, Strijk JE, Proper KI, van Wier MF, van Mechelen W, van Tulder MW, van der Beek AJ. A cost-effectiveness and return-on-investment analysis of a worksite vitality intervention among older hospital workers: results of a randomized controlled trial. J Occup Environ Med. 2013 Mar;55(3):337-46. doi:10.1097/JOM.0b013e31827b738e. 10.1097/JOM.0b013e31827b738e 23439274

[39] Strijk JE, Proper KI, van Mechelen W, van der Beek AJ (2013). Effectiveness of a worksite lifestyle intervention on vitality, work engagement, productivity, and sick leave: results of a randomized controlled trial. Scand J Work Environ Health.

[40] Rutanen R, Nygård C, Moilanen J, Mikkola T, Raitanen J (2014). Effect of physical exercise on work ability and daily strain in symptomatic menopausal women : a randomized controlled trial. Work.

[41] Hughes SL, Seymour RB, Campbell RT, Shaw JW, Fabiyi C, Sokas R (2011). Comparison of two health-promotion programs for older workers. Am J Public Health.

[42] Merrill R.M, Aldana S.G, Tonya P, Howe G, Anderson DR, Whitmer R.W. The Impact of Worksite Wellness in a Small Business Setting. J Occup Environ Med. 2011;53(2):127–31. doi:10.1097/JOM.0b013e318209e18b. 10.1097/JOM.0b013e318209e18b 21270650

[43] Strijk JE, Proper KI, van der Beek a J, van Mechelen W. A worksite vitality intervention to improve older workers’ lifestyle and vitality-related outcomes: results of a randomised controlled trial, J Epidemiol Community Heal. 2012;66(11):1071–8. 10.1136/jech-2011-200626 PMC3465836 22268128

[44] Chen M-M, Tsai a C, Wang J-Y. The effectiveness and barriers of implementing a workplace health promotion program to improve metabolic disorders in older workers in Taiwan, Glob Health Promot. 2014;0:1–9. 10.1177/1757975914555341 25355494

[45] Cook RF, Hersch RK, Schlossberg D, Leaf SL. A Web-based health promotion program for older workers: randomized controlled trial, J Med Internet Res. 2015;17(3):e82. 10.2196/jmir.3399 PMC4390614 25830503

[46] Brooke E, Taylor P, Mcloughlin C, Di Biase T. Managing the working body: active ageing and limits to the ‘flexible’ firm,. Ageing and Society. 2013;33(8):1295–1314. doi:10.1017/S0144686X12000426.

[47] Fogg NP, Harrington PE, Mcmahon BT (2012). The triumph of older workers during the great recession: implications for employers and disability policy. J Vocat Rehabil.

[48] Gabrielle S, Jackson D, Mannix J (2008). Adjusting to personal and organisational change: views and experiences of female nurses aged 40–60 years. Collegian.

[49] Longo J (2013). Bullying and the older nurse. J Nurs Manag.

[50] Malinen S, Johnston L (2013). Workplace ageism: discovering hidden bias. Exp Aging Res.

[51] Melillo Devereaux K (2013). Cognitive health and older workers. J Gerontol Nurs.

[52] Piekkola H (2006). Nordic policies on active ageing in the labour market and some European comparisons. Int Soc Sci J.

[53] Thorsen S, Rugulies R, Løngaard K, Borg V, Thielen K, Bjorner JB (2012). The association between psychosocial work environment, attitudes towards older workers (ageism) and planned retirement. Int Arch Occup Environ Health.

[54] Truxillo D.M, Cadiz D.M, Hammer L.B. Supporting the Aging Workforce: A Review and Recommendations for Workplace Intervention Research. Annual Review of Organizational Psychology and Organizational Behavior. 2015;2:351-81. doi:10.1146/annurev-orgpsych-032414-111435.

[55] Decarli C, Kawas C, Morrison JH, Reuter-Lorenz PA, Sperling RA, Wright CB (2012). Session II: mechanisms of age-related cognitive change and targets for intervention: neural circuits, networks, and plasticity. J Gerontol A Biol Sci Med Sci.

[56] Magnavita N, Garbarino S (2013). Is absence related to work stress? a repeated cross-sectional study on a special police force. Am J Ind Med.

[57] Downey AM, Sharp DJ (2007). Why do managers allocate resources to workplace health promotion programmes in countries with national health coverage?. Health Promot Int.

[58] Vanhoorne M.N, Vanachter O.V, De Ridder M.P, Occupational health care for the 21st century: from health at work to workers’ health. Int. J. Occup. Environ. Health. 2006;12(3):278–85. 10.1179/oeh.2006.12.3.278 16967837

[59] Parks K.M, Steelman L.A. Organizational wellness programs: A metaanalysis, J Occup Health Psychol. 2008;13(1):58–68. doi:10.1037/1076-8998.13.1.58. 10.1037/1076-8998.13.1.58 18211169

[60] Pegus C, Bazzarre TL, Brown JS, Menzin J (2002). Effect of the heart at work program on awareness of risk factors, self-efficacy, and health behaviors. J Occup Environ Med.

[61] Aldana SG, Greenlaw R, Diehl HA, Englert H, Jackson R (2002). Impact of the coronary health improvement project (CHIP) on several employee populations. J Occup Environ Med.

[62] Bloch MJ, Armstrong DS, Dettling L, Hardy A, Caterino K, Barrie S (2006). Partners in lowering cholesterol: comparison of a multidisciplinary educational program, monetary incentives, or usual care in the treatment of dyslipidemia identified among employees. J Occup Environ Med.

[63] CDC National Center for Chronic Disease Prevention and Health Promotion. Public Health Strategies for Preventing and Controlling Overweight and Obesity in School and Worksite Settings. A Report on Recommendations of the Task Force on Community Preventive Services. MMWR Morbidity and Mortality Weekly Report 2005; 54: RR-10. Accessible at: http://www.cdc.gov/mmwr/pdf/rr/rr5410.pdf. 16261131

[64] Chapman L. S. Expert opinions on ‘best practices’ in worksite health promotion (WHP). Am J Health Promot. 2004;18(6):1–6. 10.4278/0890-1171-18.6.TAHP-1 15293931

[65] Magnavita N SA, De Lorenzo G (2014). Health promotion in the workplace. Med Lav.

